# Altered social behavior and ultrasonic communication in the dystrophin-deficient *mdx* mouse model of Duchenne muscular dystrophy

**DOI:** 10.1186/s13229-015-0053-9

**Published:** 2015-10-29

**Authors:** Rubén Miranda, Flora Nagapin, Bruno Bozon, Serge Laroche, Thierry Aubin, Cyrille Vaillend

**Affiliations:** CNRS, Neuroscience Paris Saclay Institute, UMR 9197, Orsay, 91405 France; Univ Paris-Sud, UMR 9197, Orsay, 91405 France; Present address: Department of Psychobiology, Universidad Complutense de Madrid, Ciudad Universitaria, 28040 Madrid, Spain

**Keywords:** Dystrophin, Dystrophinopathies, Dystroglycan, Autism, Executive functions, Ultrasonic vocalizations, Social behavior, Neuroligin, Mdx mice

## Abstract

**Background:**

The Duchenne and Becker muscular dystrophies (DMD, BMD) show significant comorbid diagnosis for autism, and the genomic sequences encoding the proteins responsible for these diseases, the dystrophin and associated proteins, have been proposed as new candidate risk loci for autism. Dystrophin is expressed not only in muscles but also in central inhibitory synapses in the cerebellum, hippocampus, amygdala, and cerebral cortex, where it contributes to the organization of autism-associated trans-synaptic neurexin-neuroligin complexes and to the clustering of synaptic gamma-aminobutyric acid (GABA)_A_ receptors. While brain defects due to dystrophin loss are associated with deficits in cognitive and executive functions, communication skills and social behavior, only a subpopulation of DMD patients meet the criteria for autism, suggesting that mutations in the dystrophin gene may confer a vulnerability to autism. The loss of dystrophin in the *mdx* mouse model of DMD has been associated with cognitive and emotional alterations, but social behavior and communication abilities have never been studied in this model.

**Methods:**

Here, we carried out the first in-depth analysis of social behavior and ultrasonic communication in dystrophin-deficient *mdx* mice, using a range of socially relevant paradigms involving various degrees of executive and cognitive demands, from simple presentation of sexual olfactory stimuli to social choice situations and direct encounters with female and male mice of various genotypes.

**Results:**

We identified context-specific alterations in social behavior and ultrasonic vocal communication in *mdx* mice during direct encounters in novel environments. Social behavior disturbances depended on intruders’ genotype and behavior, suggesting alterations in executive functions and adaptive behaviors, and were associated with selective alterations of the development, rate, acoustic properties, and use of the ultrasonic vocal repertoire.

**Conclusions:**

This first evidence that a mutation impeding expression of brain dystrophin affects social behavior and communication sheds new light on critical cognitive, emotional, and conative factors contributing to the development of autistic-like traits in this disease model.

**Electronic supplementary material:**

The online version of this article (doi:10.1186/s13229-015-0053-9) contains supplementary material, which is available to authorized users.

## Background

Studies of genetic factors underlying the comorbid diagnosis of autism in genetic syndromes associated with intellectual disability, such as in the Duchenne muscular dystrophy (DMD) and milder Becker condition (BMD), may open new routes to understand the common biological mechanisms and intricate combination of multiple genes and mutations involved in the complex etiology of autism [1]. DMD and BMD are recessive X-linked neuromuscular diseases mainly affecting males and caused by mutations in the *dmd* gene that encodes the 427-kDa cytoskeleton-associated dystrophin protein (Dp427) [2]. Both syndromes are associated with non-progressive cognitive deficits, leading to intellectual disability in about 30 % of the patients [3]. The prevalence rate of autism spectrum disorders (ASD) is significantly increased (3 to >10 %) in DMD/BMD compared to the general population (<1.5 %) (http://www.cdc.gov/) [4–7]. This may be attributed to the loss of dystrophin within a functional network of brain structures including the cerebellum, hippocampus, amygdala and associative cortical areas [2, 8], which is reminiscent of the integrated circuit proposed as the neural substrate of ASD [1, 9]. Social behavior problems and poor facial affect recognition have been described in DMD children [10, 11], as well as reading and language retardation and oral phonological deficiency [12], showing that brain dystrophin loss alters both social behavior and communication.

The phenotypic heterogeneity in dystrophinopathies likely relies on individual differences in genetic background and on the variety of mutation profiles within the *dmd* gene, which may lead to the sole loss of brain full-length dystrophin (Dp427) or to a cumulative loss of shorter dystrophins encoded by distinct internal promoters, such as Dp140 and Dp71 [13]. While the presence of mental retardation in one third of DMD patients is closely related to distal mutations affecting expression of C-terminal forms of dystrophin (e.g., [13]), no clear genotype–phenotype relationship was reported for the comorbid diagnosis of ASD, and patients with altered social behavior and communicative skills do not necessarily display mental retardation [6, 10, 11, 14]. However, one patient with DMD and autism was shown to carry a submicroscopic deletion encompassing exons 12–25 of the dystrophin transcript, suggesting that the loss of Dp427 is sufficient to induce vulnerability to autism [15]. The dystrophin-associated complex links the actin-based cytoskeleton to the extracellular matrix in both the muscle and brain, where it interacts with specific membrane receptors and ion channels [16]. Brain alterations associated with Dp427 loss are mainly located at the synaptic level and involve impaired gamma-aminobutyric acid (GABA)ergic function and excitation/inhibition balance; key mechanisms also implicated in ASD [17]. Dystroglycan, a central component of dystrophin complexes interacts with the autism-associated trans-synaptic neurexin-neuroligin complex [18–20], suggesting a putative mechanism underlying alterations in social behavior and/or communication in DMD. In the present study, we have characterized alterations in social behavior and ultrasonic communication in the *mdx* mouse model of DMD, which presents a nonsense point mutation (C-to-T transition) in exon 23 of the *dmd* gene that aborts full-length dystrophin (Dp427) expression [21]. We used a combination of behavioral and bioacoustics measures previously validated as relevant approaches to characterize autistic traits in mice. Parameters have been quantified in a range of socially relevant paradigms involving various degrees of executive/cognitive demand, from simple presentation of sexual olfactory stimuli to social choice situations and direct encounters with female and male mice of various genotypes, which enabled the identification of critical cognitive, emotional, and conative factors contributing to the phenotype.

## Methods

### Animals

C57BL/10ScSn-Dmd^*mdx*^/J mutant males (*mdx*) and littermate wild-type (WT) controls were bred in our laboratory [22]. Male siblings were kept in groups (three to six mice) under a 12-h light–dark cycle (light on 7:00 a.m.) with food and water ad libitum*.* A first cohort of 5-month-old male mice (*mdx* = 13; WT = 10) was placed in individual cages for 1 week and then successively tested for social interaction with females, response to females’ urine and cage bedding, social interaction with males, investigation of anesthetized females, and social choices in the three-chamber test, with at least 48-h intervals between tests (total duration of isolation period 4–5 weeks). A second cohort of 3-month-old male mice (*n* = 15 per genotype) was placed in individual caging for 2 months to increase the level of territorial aggressiveness before being tested in the resident–intruder agonistic paradigm. In both experiments, mice were 5 months old at the start of testing. Adult control mice (>3 months) used for encounters or for bedding/urine collection were obtained from Harlan Laboratories (C57BL/10ScSn01aHsd) and Janvier Europe (C3H and balb/c, all males) and were reared in groups throughout. Mice pups aged 3 to 9 postnatal days (PNDs) from four different litters (*mdx* = 7; WT = 15) were tested for neonatal ultrasonic vocalizations (USVs). Experiments were conducted blind to genotype and in accordance with the European Communities Council Directive of 24 November 1986 (CEE 86/609/EEC), EU Directive 2010/63/EU and French National Committee (87/848), and following guidelines of the local animal facility (Direction Départementale de la Protection des Populations, DDPPV-France, agreement # B91-471-104).

### Recording of ultrasonic vocalizations

Mouse ultrasonic calls were recorded in mouse pups following mother separation and in adult mice during interaction tests and exposure to olfactory stimuli as in previous studies [23–27]. For recordings of USVs in neonates, mdx and WT breeding pairs were housed in standard Plexiglas cages (20 × 30 cm). The day of birth was considered PND0 and pups were tested every day from PND3 to PND9. At the time of testing, each pup was separated from its mother and placed into an empty cage (20 × 30 cm) inside a sound-attenuating Styrofoam box to assess USVs for 5 min. Room temperature was kept at 22 ± 1 °C. Pups were placed back into their home cage after testing. Individuals were tagged by toe clipping to allow identification during successive days. Data from all individual pups were pooled within each genotype, as no obvious sex-specific call patterns were observed. For adult mice, USVs were recorded in a range of experimental conditions as described below in the “Behavioral testing” section, except in the resident–intruder test performed in residents’ home cage, due to the noise associated with the pursuits and agonistic behaviors, and in the social approach in the three-chambered test, due to the size and architectural constraints of the apparatus.

USVs were recorded with a condenser ultrasound microphone (Avisoft Bioacoustics, Germany; CMPA-P48/CM16, frequency response ±3 dB within the range 10–180 kHz) suspended 15 cm above the cage and connected to a TASCAM HD-P2 digital recorder. Vocalizations were sampled at 192 kHz with a 16-bit dynamic. Recordings were analyzed with the Avisoft SASLab Pro (v4.40) signal processing software. Spectrograms were thus generated (fast Fourier transform (FFT)-length of 1024 points, overlap of 75 %, 100 % Frame, Hamming window) to follow the frequency modulation. A high-pass FFT filter (cutoff frequency of 25 kHz) was applied to reduce the background noise. A call, or syllable, was defined as a unit of sound separated by silence from other sound units that may consist of one or more “notes” or continuous markings on a sonogram [23]. A sequence, or bout, was defined as a succession of at least two calls separated by silent intervals less than 200 ms. Number and mean duration of calls and sequences were quantified, and the total time spent calling was computed by summing durations of each call. The call peak frequency (Pfreq) was defined as the frequency of maximum amplitude in the spectrum, while the call peak amplitude (Pamp) corresponded to the amplitude at peak frequencies at the start and end points of each call. Measures taken in repeated trials were averaged for statistical comparisons.

### Semi-automatic quantification of USV subtypes

**Fig. 1:**
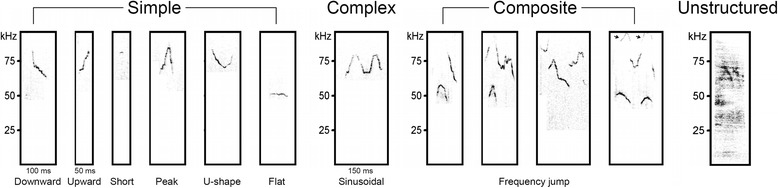
Classification of mouse ultrasonic calls. Sample images illustrate the main categories identified in mouse ultrasonic calls. *Arrows* in the last example of frequency jump USV indicate the presence of harmonic components, which have been used as an additional classification criterion. The broader (inclusive) categories grouping several call subtypes are indicated on *top* (i.e., simple, complex, and composite categories)

A qualitative detection of ultrasonic calls was performed in both pups (*N* = 21,060 calls) and adults (*N* = 79,601 calls) using a combination of custom visual basic for application (VBA)-based macros and visual inspection of sonograms (Additional file 1: Supplementary method section), which enabled identification of 10 waveform pattern categories [24, 25, 27] (see Fig. 1): *upward*: continuous frequency increase of at least 1.5 Hz per 10-ms bins, with eventually flat steps; *downward*: continuous frequency decrease of at least 1.5 Hz per 10-ms bins, with eventually flat steps; *peak*: frequency-modulated call showing continuous increase in frequency followed by a continuous decrease; *u-shape*: frequency-modulated call showing continuous decrease in frequency followed by a continuous increase; *flat*: constant frequency with no modulation >1.5 Hz per 10-ms bins; *short*: duration <10 ms; *sinusoidal* (*complex*): two or more directional changes in frequency in distinct directions; *frequency jump* (*composite*): two or more components displaying discontinuous frequency “jump (s)” on the sonographic representation but without gap on the time scale; *harmonic*: fundamental frequency slightly modulated combined with an amplified harmonic component. Because harmonics were observed in different call categories, each call was first assigned to one of the above categories and then further labeled as “harmonic” or “non-harmonic”; *unstructured*: without main sound component and frequency shape that could be assimilated to any of the other categories. Calls were then grouped in a reduced number of broader (inclusive) categories defined as: *simple* (i.e., upward, downward, flat, short, peak, and u-shaped), *complex* (i.e., sinusoidal), and *composite* (i.e., frequency jump).

To detect and classify calls, an automatic threshold-based detection of calls was first performed by extracting time/frequency data every 10 ms for each call (Avisoft SASLab Pro v4.40). Data were then imported into Excel software (Microsoft corp.) and a custom VBA macro was run for automatic classification of calls: Within each call, each 10-ms interval was assigned to a qualitative value representing ascending, descending, or flat changes in frequency (≥1.5 Hz). For the total duration of a call, the macro then integrated these 10-ms sequences of qualitative information to determine the category to which the call belongs (upward, downward, flat, peak, u-shaped, or sinusoidal). Short calls were simply identified based on their duration. Automatic threshold detection was not accurate to identify unstructured, harmonic, and frequency jump (i.e., composite) calls (Fig. 1), or in conditions of low signal to noise ratio. In such conditions, an accurate classification was achieved manually and calls were assigned to adequate categories using the *interactively* (*section labels*) function of Avisoft software. Data derived from manual inspection of sonograms were transferred into Excel software and combined with previous automatically classified data, using a second VBA-based custom macro. Using the start points of each call as a reference, this second macro overwrote and corrected previous automatic classification data with manually defined categories when appropriate. A detailed description of the classification strategy is provided in the Additional file 1 (supplementary method section), and VBA-based macros are available upon request to corresponding authors.

### Behavioral testing

#### Male–female social interaction

Adult male mice of each genotype were confronted with control (WT) females for 3 min during four consecutive days, to ensure that all subjects reliably emitted USVs. Each test male was confronted with a different female in every trial. For each testing day, test males were first familiarized to the test cage (transparent Plexiglas box, 20 × 30 × 14 cm, with floor filled with fresh bedding) for 30 min, an unknown control female was introduced, and social interaction was recorded for 3 min. Female estrous stage was verified each day after behavioral testing by vaginal smear cytology. Behavioral responses were analyzed during the first session. Behavioral responses were videorecorded, and the sniffing or snout contacts elicited by the resident males were quantified manually using event-recorder keys in ANY-maze software (Stoelting, USA). The latency of first contact, mounting attempts, frequency, and duration of contacts were analyzed. Contacts were classified as orofacial (directed towards the head/neck/mouth area), orogenital (towards the anogenital area), or directed towards other body parts (flank area).

#### Male exposure to female urine

Test mice were first acclimatized to the testing room and cage for 30 min and then tested during two consecutive days. Each daily session consisted in three consecutive trials (5-min duration) with a 5-min inter-trial interval, consisting in the successive presentation of female mouse urine (trial 1), distilled water (trial 2), and mouse urine from a different female (trial 3). A 20-μl drop of urine or distilled water on a cotton swab was placed in the testing arena hanging from a telescopic clamp close to the microphone (about 15 cm above the floor). Fresh urine was previously collected from WT female donors by holding them by the scruff onto a metal grid, above a clean aluminum foil. In case handling was not sufficient to stimulate urination, the mouse ventral area was gently stroked in the anteroposterior direction [28]. The urine on the foil was immediately pipetted into Eppendorf tubes for storage at −80 °C and classified according to individual estrous stage (determined by vaginal smear cytology after urine collection). On the day of testing, frozen urine from proestrous-estrous females (matching peak levels of estradiol and progesterone) was thawed at room temperature and pipetted onto clean cotton swabs. Mice were videotracked (ANY-maze, Stoelting, USA) to analyze the distance travelled and the frequency and duration of mobile episodes. Sniffing of the cotton swab was manually quantified using event-recorder keys in ANY-maze and the percentage time sniffing compared between genotypes.

#### Male exposure to female cage bedding

The test mice were first acclimatized to the testing room and cage for 30 min. Then, a daily session consisted in two consecutive 3-min trials. During the first trial, bedding from a females’ cage (approximately 25 ml, cage unchanged for 1 week) was presented in one of two identical recipients (4 × 4 × 1 cm) placed at opposite corners of the testing cage. In trial 2, the previously empty recipient was filled with new bedding collected from a different females’ cage. Location of olfactory stimuli used in trials 1 and 2 was balanced across animals. The distance travelled and the frequency and duration of mobile episodes were quantified. The frequency and duration (expressed as percentage time) of bedding recipients sniffing were manually scored using event-recorder keys in ANY-maze (Stoelting, USA).

#### Male exposure to anesthetized female

Following acclimatization to the test cage as above, the test mice were confronted to an anesthetized control female (anesthesia: IP injections of 100 mg/kg ketamin and 12 mg/kg xylazin) during two 3-min successive trials performed on the same day with an inter-trial interval of 2 min. A different female was introduced in the test cage in each trial. The frequency and duration (percentage time) of contacts performed by the test mice were manually quantified using event-recorder keys in ANY-maze (Stoelting, USA).

#### Male–male reciprocal social interaction

Social interaction was assessed as for the male–female interaction test above. Resident male mice were confronted to unknown male intruders (either of a WT or *mdx* genotype) for 3 min on two consecutive days. Behavioral responses were manually scored in both resident and intruder mice including the latency of the first contact, number of dominance responses (resident putting paws onto intruders back), frequency and duration (percentage time) of pursuits, orofacial sniffing, orogenital sniffing, and body sniffing. No mounting, fighting, and wrestling behaviors were observed.

#### Social approach behavior in a three-chamber test

Apparatus and paradigm have been previously described [29]. The apparatus was a rectangular three-chambered box fabricated from clear polycarbonate. Dividing walls with manual retractable doorways (width 10 cm; height 20 cm) enabled access into each chamber (20 × 40 × 22 cm). The box was cleaned with alcohol, and fresh bedding was added between trials. The middle chamber was used to confine test mice at the beginning of test trials, while each of the two side chambers contained an empty transparent polycarbonate tube (8 cm in diameter) in which social (stranger mouse) and nonsocial (object) stimuli could be confined. These confinement tubes were drilled with holes (1-cm diameter; 2-cm spaced) to allow limited contact with the confined stimulus. Control male mice used as social stimuli (strangers) were familiarized to the confinement tubes during 4 days (30 min per day) before the start of the experiment. Then, testing consisted of three trials: For trial 1 (habituation), the test mouse was placed in the middle chamber for 2 min, the doorways were then opened, and the mice could freely explore the test box for 10 min after which they were confined in the middle chamber for 30 s before proceeding with the next trial. During trial 2 (sociability), an unknown mouse (stranger 1) was introduced in one of the confinement tubes in one side chamber, whereas an object made of Lego® pieces was enclosed in the other tube in the opposite side chamber. Location of stranger 1 in the left or right side chamber was balanced across subjects. Upon door re-opening, the test mouse was allowed to explore the test box and tubes for 10 min and was then confined again in the middle chamber before the next trial. During trial 3 (preference for social novelty), the object present in trial 2 was replaced by a novel unfamiliar mouse (stranger 2), and the test mouse was then given the possibility to interact with both stranger 1 and stranger 2 for 10 min. Behavior was videotracked during all trials (ANY-maze software) to analyze general activity (distance travelled, rearing and leanings, self-grooming, periods of activity and inactivity). Social behavior was reflected by the percentage time spent and number of entries in the side chambers containing the confined stimuli, as well as by the frequency and duration (percentage time) of sniffing episodes directed against the confinement tubes.

#### Resident–intruder test

The protocol was adapted from previously described methods [30]. Test mice of both genotypes (*mdx* and WT littermates) were initially changed to individual caging (20 × 30 cm cages) for 2 months without bedding change. Three distinct strains of mice were used as intruders, based on their known differences in social behavior: male C57BL/10 mice aged 3–4 months, which were of the same genetic background as the resident test mice; males of the C3H strain, known as non-aggressive mice; and males of the balb/c strain known for their high intraspecific aggressiveness [31]. Each test mouse was successively confronted to these three types of intruders within a 2-week period (5-day intervals between trials).

Behavior was videorecorded for 6 min after placing the intruder in the resident’s home cage. The following behavioral parameters were coded manually using event-recorder keys (ANY-maze software): contacts initiated by the resident, contacts initiated by the intruder, fights, pursuits, rearings, dominant behavior (putting a paw on the body of the other mouse and/or mounting), as well as periods of immobility either during or between social contacts. The specific nature of the contacts was further specified as orofacial, orogenital, and body sniffing.

### Statistical analyses

Data presented as means ± SEM correspond to averages of responses expressed in successive trials, as no significant variations in performance were detected across repetitive trials in a preliminary analysis using repeated measures analysis of variance (ANOVA). Ultrasonic calls and behaviors were analyzed using two-way ANOVAs with one between-subject factor (genotype: WT or *mdx*) and one dependent variable or within-subject factor when appropriate (postnatal day, task, type of contact, chamber), followed by Tukey post hoc tests. Correlations were calculated using the Pearson correlation coefficient. *P* values <0.05 were considered statistically significant. The two-tailed Kolmogorov-Smirnov (KS) test was used to analyze the distribution of call duration (significance threshold: *p* < 0.005).

## Results

### Social approach behavior in the three-chambered box

**Fig. 2:**
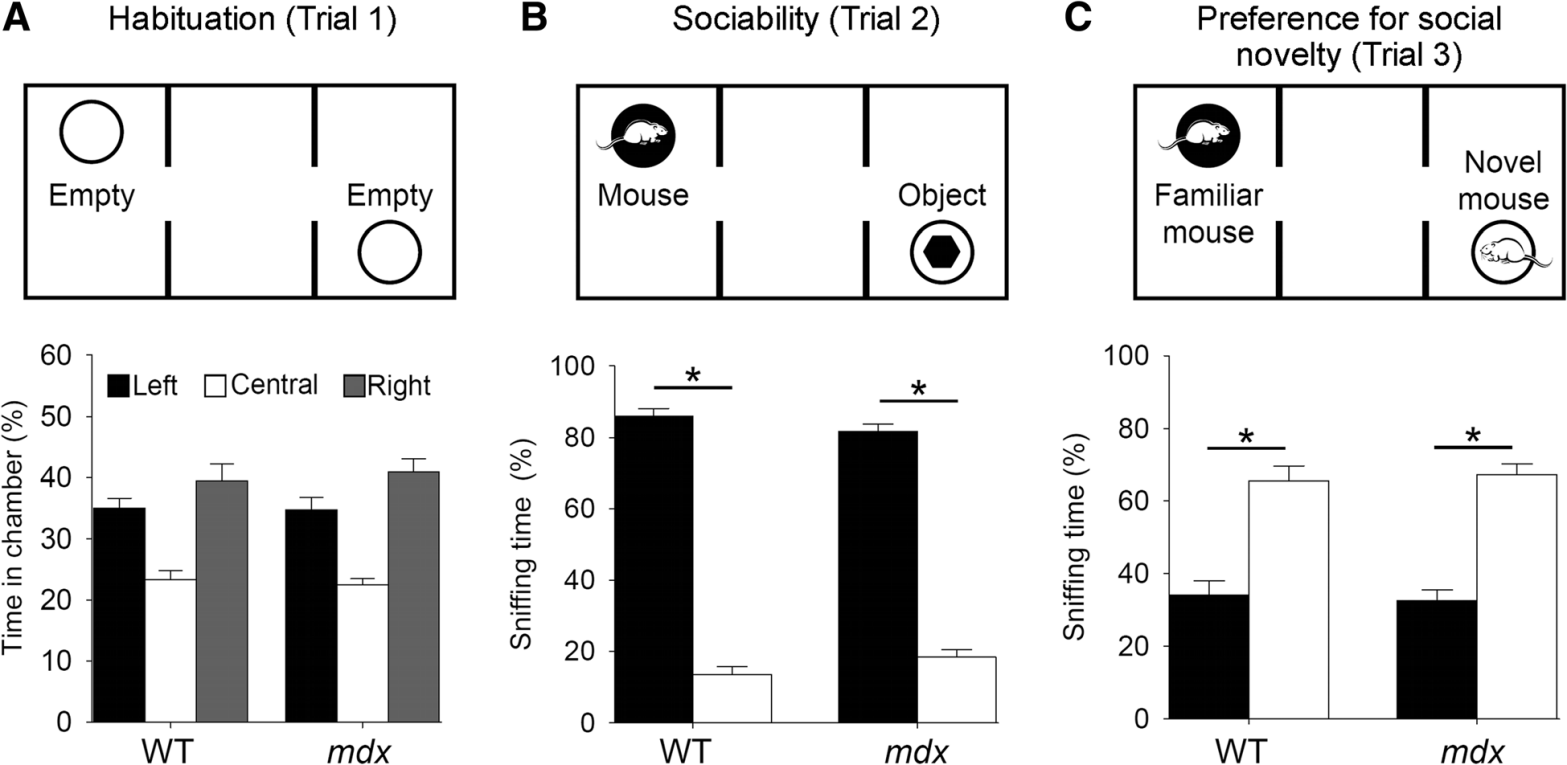
Social behavior in the three-chambered test. *Top panels* show the experimental setup during (**a**) exploration of the apparatus that only contained empty confining tubes (habituation; trial 1), (**b**) choice between a stranger mouse and an object (sociability; trial 2), and (**c**) choice between a familiar and a novel mouse (preference for social novelty; trial 3). *Histograms* represent exploratory behavior during the three trials, expressed as percent time spent in central and side (left, right) chambers during habituation; (**a**) percent time spent sniffing the tubes containing the mouse (*black bars*) and the object (*white bars*) during the sociability test and (**b**) percent time spent sniffing the familiar (*black bars*) and novel mice (*white bars*) during the social novelty test; (**c**) *p < 0.05

Spontaneous activity was significantly lower in *mdx* than in WT mice during habituation (Table 1; trial 1; *p* < 0.05). Both genotypes explored the side chambers more than the central zone (percentage time in chamber, *p* < 0.05), with no place preference for any side chamber (Fig. 2a). Sociability (trial 2) was comparable between genotypes with a clear preference for the chamber containing the social stimulus (chamber effect: *p* < 0.05; genotype effect and genotype × chamber interaction: *p* > 0.05; Fig. 2b). In trial 3 (preference for social novelty; Fig. 2c), mice spent more time sniffing the novel mouse than the familiar mouse (percentage time sniffing, *p* < 0.05) and no significant genotype effect was detected, demonstrating normal preference for social novelty and short-term social memory in *mdx* mice.

**Table 1:** Behavioral activity during habituation in the three-chambered box. The distance run and time spent mobile/active were significantly lower in *mdx* than in wild-type (WT) mice. However, rearings and self-grooming activity (*p* > 0.05) were unaffected

	WT (*n* = 10)	*mdx* (*n* = 13)	*P* values
Distance run (m)*	47.93 ± 2.36	40.32 ± 1.37	0.008
Time mobile (s)*	556.84 ± 7.23	536.38 ± 6.26	0.044
Time active (s)*	564.27 ± 6.81	543.35 ± 5.62	0.026
Rearings (*n*)	83.70 ± 8.65	95.15 ± 6.43	0.289
Self-grooming (*n*)	2.20 ± 0.61	2.08 ± 0.38	0.860
Grooming duration (s)	8.44 ± 2.31	6.86 ± 1.09	0.512

*p < 0.05

### Reciprocal social interactions

#### Social interaction in a novel environment

**Fig. 3:**
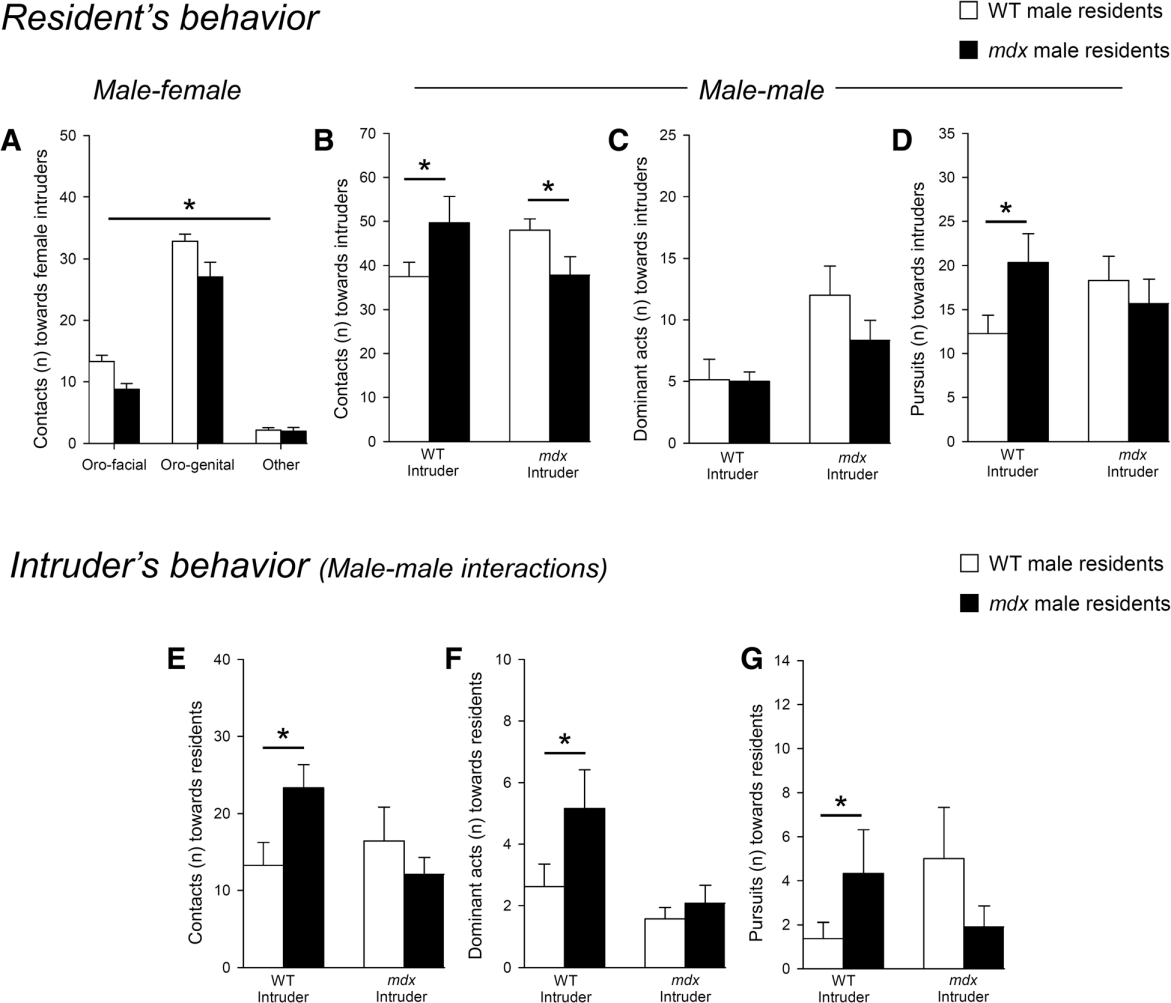
Reciprocal social interactions in a novel cage. **a** Male–female interactions expressed by the number of orofacial, orogenital, and body contacts initiated by WT and *mdx* resident mice towards control intruder females. **b**–**d** Male–male interactions expressed by the behavioral responses initiated by WT (*white bars*) and *mdx* resident male mice (*black bars*) during interactions with WT and *mdx* male intruders (intruder’s genotype indicated on the *X*-axis). Histograms show the number of contacts (**b**), dominant acts (**c**), and pursuits (**d)** performed by resident test mice. **e**–**g** Behaviors initiated by WT and *mdx* intruder male mice during interactions with WT and *mdx* male residents. *Histograms* show the number of contacts (**e**), dominant acts (**f**), and pursuits (**g**) performed by the intruders. *p < 0.05

The social behavior of *mdx* resident males differed depending on the sex and genotype of the intruder. As shown in Fig. 3a, *mdx* mice initiated fewer contacts with females as compared to WT mice (*p* < 0.005; Fig. 3a), which was not associated with changes in specific types of contacts. In male–male encounters (Fig. 3b–d), the *mdx* resident mice also displayed fewer contacts towards *mdx* male intruders (*p* < 0.05) but conversely initiated more contacts than control residents when confronted to WT intruders (*p* < 0.05) (Fig. 3b). In any cases, no fights were observed and the latency of the first contact (all <10 s) and the number of dominant acts initiated by resident test mice (Fig. 3c) were comparable regardless of the intruder’s genotype (*p* > 0.05). However, the increased number of contacts with WT intruders, as shown in Fig. 3b, was associated with the initiation of an increased number of pursuits directed against the WT intruders (*p* < 0.05). This was not observed when *mdx* residents were confronted with *mdx* intruders (*p* > 0.05; Fig. 3d), showing that pursuit behavior, as well as the number of contacts, varied depending on the intruder’s genotype.

Interestingly, the intruder’s behavior also varied depending on the resident’s genotype (Fig. 3e–g). The behavior of *mdx* intruders was not influenced by the resident’s genotype while, in contrast, the behavior of WT intruders varied when confronted with *mdx* residents: indeed, the WT intruders initiated more contacts (Fig. 3e), dominant acts (Fig. 3f), and pursuits (Fig. 3g) during interactions with *mdx* residents than with other WT residents (all parameters, *p* < 0.05). This suggests that *mdx* mice, when acting as passive (or receptive) residents, were showing more submissive responses compared to controls.

#### Resident–intruder agonistic interactions

**Fig. 4:**
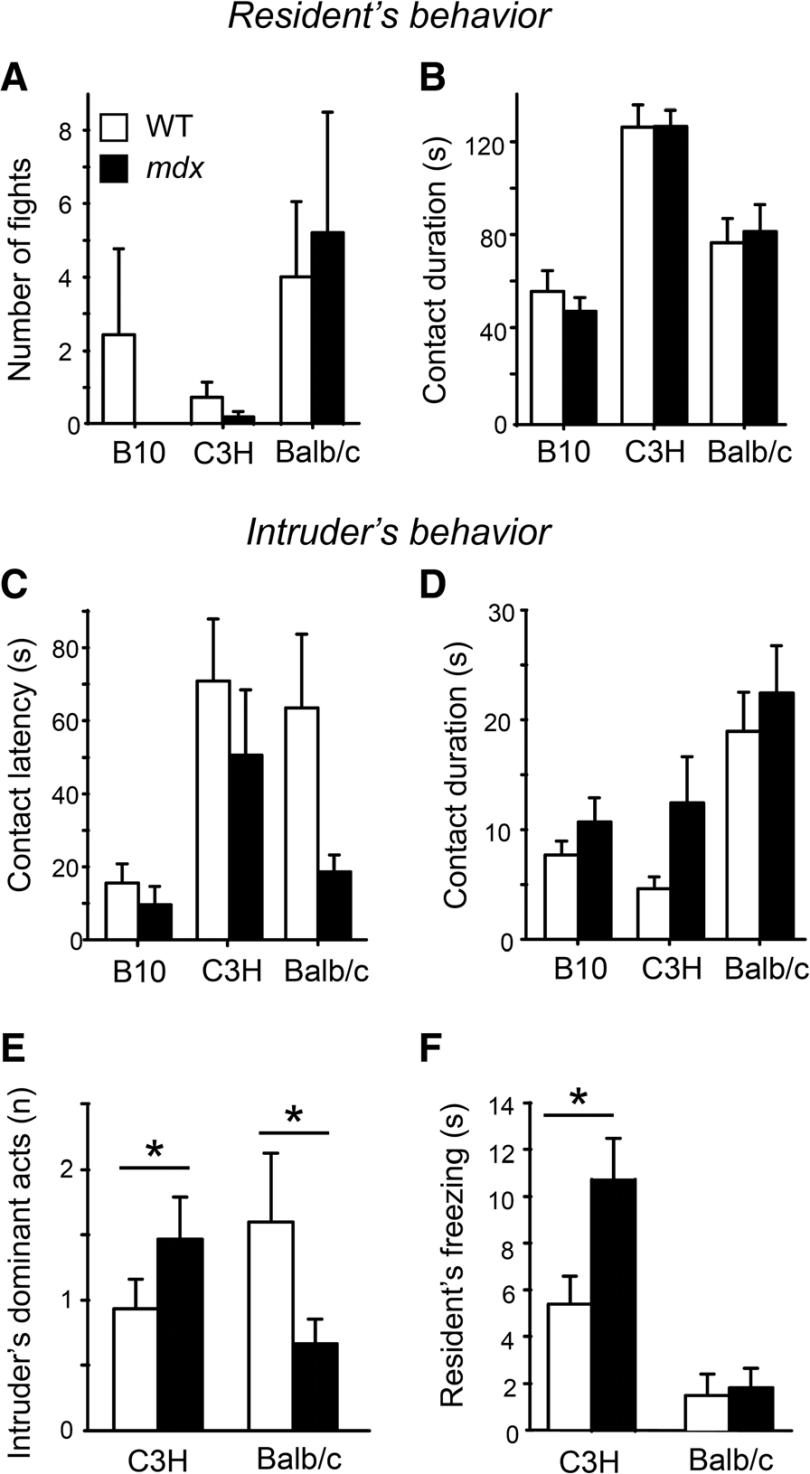
Resident–intruder agonistic interactions in resident’s home cage. **a**, **b** Number of fights (**a**) and duration of contacts (**b**) initiated by WT and *mdx* residents. There was no effect of resident’s genotype on these parameters, but note that the expression of agonistic and affiliative behaviors differed depending on the genotype of the intruder mice (e.g., less fights and longer interactions with C3H intruders). **c**, **d** Intruders’ behavior varied depending on the genotype of the resident mice, as shown by shorter latencies (**c**) and slightly longer duration of social contacts when confronted with *mdx* mice (**d**). **e** The number of dominant acts (i.e., putting a paw on the resident’s head or back) differed depending on the resident’s genotype, as illustrated by the opposite effects shown in the non-aggressive C3H intruders compared to the aggressive balb/c intruders. **f** Fear responses in resident mice, expressed as time spent freezing during social interactions, were more prominent during confrontation with non-aggressive C3H intruders and enhanced in *mdx* compared to WT resident mice. *p < 0.05

To determine whether changes in intruder’s aggressiveness influenced the variable behavior of *mdx* resident mice in the experiments above, a different group of male residents was submitted to a longer period of social isolation (2 months before testing) and forced encounters with intruders of three distinct genotypes were performed in the resident’s home cage (instead of a novel cage) to increase agonistic interactions. Fights were scarce with C57BL/10 and C3H intruders but more consistently observed with balb/c intruders (Fig. 4a). Although pursuits and fights were mainly initiated by residents, the intensity of aggressiveness was thus dependent on the intruders’ behavior. Still, the incidence of fights was relatively low, which allowed reliable recording of social behaviors. Self-grooming episodes were scarce and very short (<4 s; no significant genotype effect). In this experiment, the intruders’ genotype also influenced the quantity of social interactions initiated by residents, as revealed by significant intruder’s genotype × dependent variable interactions (contact latency: *p* < 0.05; number: *p* < 0.01; duration: *p* < 0.001). Figure 4b shows the longer duration of contacts in residents of both genotypes in presence of low-aggressiveness C3H intruders (*p* < 0.001).

Conversely, intruder mice displayed shorter latencies to interact with *mdx* as compared to WT residents (genotype: *p* < 0.05; genotype × intruder interaction: *p* = 0.3; Fig. 4c) associated with a slightly longer duration of contacts (*p* = 0.09; Fig. 4d). To better understand why the intruders behaved differently when confronted with *mdx* residents, we compared the quality of the social interactions when intruders were of the C3H and balb/c genetic backgrounds during the first 3 min of testing (Table 2). There was an increased expression of a specific set of behavioral responses, such as orofacial contacts, pursuits, dominant acts, and freezing behavior, when resident mice were confronted with C3H intruders as compared with interactions involving balb/c intruders. Moreover, the non-aggressive C3H intruders displayed more dominant acts towards *mdx* mice, while the more aggressive balb/c mice directed dominant acts preferentially towards WT residents (Fig. 4e). Periods of immobility (freezing), which likely reflected fear responses, were more frequent and longer during physical contacts in *mdx* residents compared to WT, while the quantity of rearings was conversely decreased (Table 2). Interestingly, immobility was comparable between genotypes during periods with no physical contacts, suggesting that the enhanced freezing in *mdx* mice was selectively triggered by social contact. Enhanced fear/defensive responses in *mdx* mice likely have favored intruder’s dominant behavior, as freezing was more prominent when *mdx* mice interacted with the C3H intruders (Fig. 4f), which also showed the largest amount of dominant behaviors towards *mdx* mice (Fig. 4e).

**Table 2:** Resident–intruder agonistic interactions. Behavioral parameters are grouped in three categories: Quality of contact, aggressive behaviors, and other behavioral responses. Only the significant statistical effects are shown (*p* values; ANOVAs with repeated measures) to highlight the effects of intruder’s genetic background (within-subject factor: C3H versus balb/c) and resident’s genotype (between-subject factor: *mdx* versus WT). The direction of the genotype differences is indicated in brackets

Behavioral responses	Effect of intruder’s genetic background	Effect of resident’s genotype	Intruder × resident interaction
Quality of contact			
Orofacial (*n*)	*p* < 0.001 (C3H > balb)	–	–
Orofacial (duration, s)	*p* < 0.001 (C3H > balb)	–	–
Orogenital (*n*)	–	–	–
Orogenital (duration, s)	–	–	–
Other (*n*)	–	–	–
Other (duration, s)	–	–	–
Aggressive behaviors			
Pursuits (*n*)	*p* < 0.05 (C3H > balb)	–	–
Pursuits (duration, s)	–	–	–
Resident dominant acts (*n*)	*p* = 0.01 (C3H > balb)	–	–
Resident dominant acts (duration, s)	*p* < 0.001 (C3H > balb)	–	–
Intruder dominant acts (*n*)	–	–	*P* < 0.05
Intruder dominant acts (duration, s)	–	–	*p* = 0.09, NS
Other behavioral responses			
Rearings (*n*)	–	*p* < 0.05 (*mdx* < WT)	–
Rearings (duration, s)	–	*P* < 0.05 (*mdx* < WT)	–
Freezing during interaction (*n*)	*p* < 0.001 (C3H > balb)	*P* < 0.05 (*mdx* > WT)	–
Freezing during interaction (duration, s)	*p* < 0.001 (C3H > balb)	*P* < 0.05 (*mdx* > WT)	*P* < 0.05
Freezing apart from interaction (*n*)	*p* < 0.05 (C3H > balb)	–	–
Freezing apart from interaction (duration, s)	–	–	–

### Exposure to olfactory sexual stimuli

Female olfactory stimuli and the presence of irresponsive (anesthetized) females elicited comparable behavioral approaches in *mdx* and WT mice. Both genotypes spent more time sniffing anesthetized females than female cage bedding and urine (task effect: *p* < 0.001; genotype and interaction effects: *p* > 0.05; Additional file 2: Figure S1).

### Ultrasonic vocalizations in pups

**Fig. 5:**
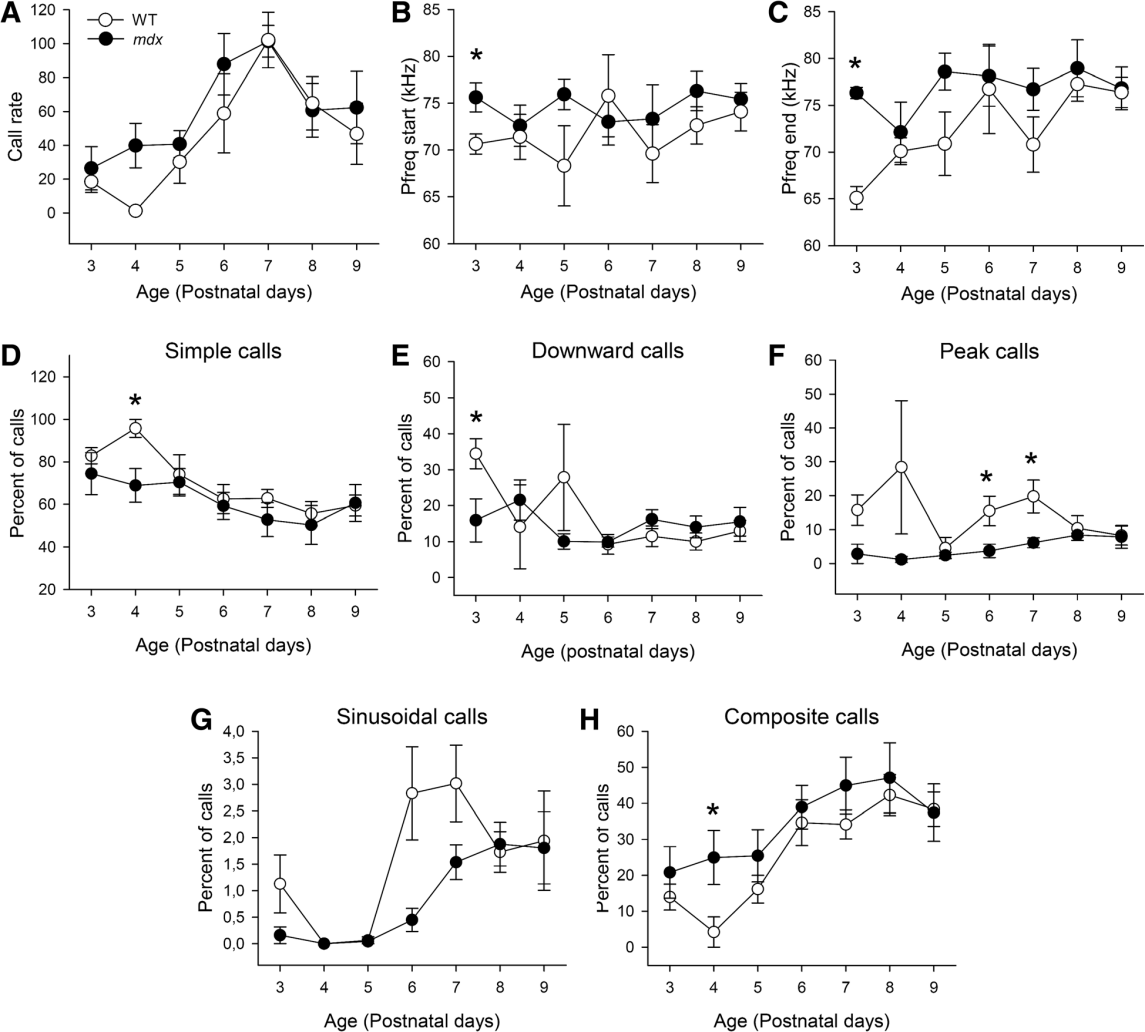
Isolation-induced ultrasonic vocalizations in pups. **a** Call rate recorded in the same groups of WT (*white circles*) and *mdx* (*black circles*) pups during postnatal days (PNDs) 3 to 9. Note that both genotypes emitted the highest amount of calls at PND7. **b**, **c** Call peak frequency (Pfreq) measured at call start (**b**) and end (**c**) points. Note the enhanced peak frequency at call start point in *mdx* pups at PND3. **d**–**h** Postnatal variations in the vocal repertoire showing transient genotype differences at specific PNDs for the percent of simple (**d**), downward (**e**), peak (**f**), sinusoidal (**g**), and composite calls (**h**). *p < 0.05

Both genotypes showed a similar developmental curve for the number of isolation-induced USVs (Fig. 5a). The number of calls, sequences, calls per sequence, as well as the call amplitude and duration and time spent vocalizing were comparable between genotypes (all parameters; *p* > 0.05). However, call peak frequencies were higher in *mdx* than WT mice at PND3 (*p* < 0.05; Fig. 5b, c), suggesting an early but transient alteration in USV properties in neonate *mdx* mice.

Most isolation calls were classified (Fig. 1) in the simple-type category, while composite and complex types were less represented. At PND4, *mdx* pups produced fewer simple (*p* < 0.05; Fig. 5d) and more composite calls compared to WT pups (*p* < 0.05; Fig. 5h), while they produced fewer downward calls at PND3 (*p* < 0.05; Fig. 5e) and fewer peak calls during PND6 and PND7 (*p* < 0.05; Fig. 5f). Overall, *mdx* mice tended to produce fewer simple and sinusoidal calls (Fig. 5d, g) but more composite calls (Fig. 5h), suggesting transient alterations in the use of ultrasonic vocal repertoire during postnatal development.

### Ultrasonic vocalizations in adults

**Fig. 6:**
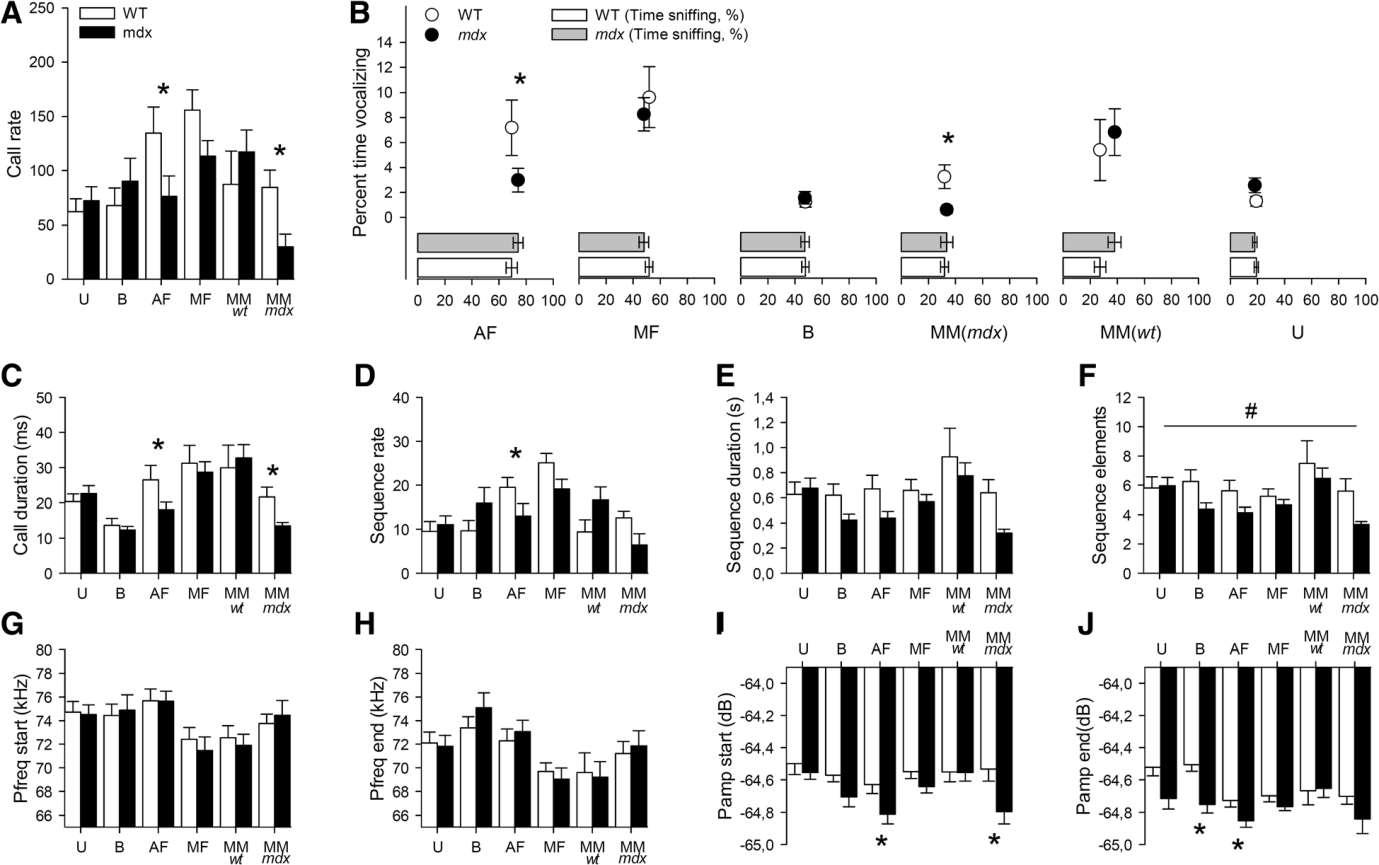
Ultrasonic vocalizations in adult mice. **a** Call rate in response to distinct olfactory and social stimuli. **b** Percent time vocalizing (*Y-*axis and *circle symbols*; WT: *white circles*, *mdx*: *black circles*) and time spent sniffing the stimuli (*X-*axis and *horizontal histograms*; WT: *white bars*, *mdx*: *gray bars*) in each test, as indicated below the *X*-axis. Tests are ordered from left to right from highest to lowest sniffing activity recorded in WT mice. *Horizontal position of circles* corresponds to the mean time sniffing. Overall, the largest amount of calls emitted by adult male mice was recorded during reciprocal interactions with freely moving females, then with anesthetized female and male–male interaction. Long time spent vocalizing was not systematically correlated with long time spent investigating stimuli. For example, exposition to females’ bedding resulted in large amounts of exploration (>40 % time sniffing) but relatively short time spent vocalizing compared to other testing situation associated with comparable time sniffing, such as in the male–female interaction. Ordering tests following the experimental test order does not reveal any progressive decrease or increase in exploration or USV production, suggesting that test order did not overcome responsiveness to social stimuli, but USV production was modulated by social context. **c**–**j** Histograms show the call duration (**c**), sequence rate (**d**), sequence duration (**e**), number of syllables or sequence elements (**f**), peak frequency at call start (**g**) and end points (**h**), and peak amplitude at start (**i**) and end points (**j**). *U* urine test, *B* bedding test, *AF* anesthetized female test, *MF* male–female interaction, *MMwt* interaction with a WT male, *MMmdx* interaction with an *mdx* male, *Pfreq* peak frequency, *Pamp* peak amplitude. *p < 0.05, Tukey post hoc test. ^#^ *p* < 0.05, main genotype effect in two-way ANOVA

In adult WT mice, the largest amounts of USV was detected during interactions with freely moving females, then in the presence of anesthetized females, then with other male encounters, and finally in response to olfactory stimuli (female cage bedding and urine) (see Fig. 6a–d). This confirms that syllable production in mice is modulated by the social context. The latency of the first call was comparable between genotypes in all conditions (*p* > 0.05). However, the call rate (Fig. 6a), percent time vocalizing (Fig. 6b), and call duration (Fig. 6c) varied depending on the experimental context and genotype (task effect: *p* < 0.001, genotype × task interaction: *p* < 0.01). The rate and duration of calls were particularly reduced in *mdx* compared to WT mice during interactions with *mdx* male intruders (MM-*mdx* in Fig. 6a–c; *p* < 0.05) and in the presence of anesthetized females (Fig. 6a–c; *p* < 0.05). There was a task-dependent reduction in the number of sequences in *mdx* mice (task effect: *p* < 0.001; genotype × task interaction: *p* < 0.005; Fig. 6d), which was particularly reduced during interaction with anesthetized females (*p* < 0.05). Duration of sequences was unaffected (Fig. 6e). In all tests except during urine presentation, *mdx* mice sequences contained fewer syllables (sequence elements; Fig. 6f) (genotype effect: *p* < 0.05; genotype × task interaction: *p* > 0.05), which was likely linked to the reduced call rate in this genotype. Call peak frequencies were globally unaffected (Fig. 6g, h). However, the peak amplitudes were less intense in *mdx* compared to WT mice during exposure to anesthetized females and female bedding and during interactions with *mdx* male intruders (*p* < 0.005; Fig. 6i, j). Analysis of the correlations between behavioral and acoustic parameters (Additional file 3: Supplementary results section) revealed that the significant correlations detected in WT mice were absent in *mdx* mice, suggesting a disorganization of the relationships between social behavior and ultrasonic communication in this model.

**Fig. 7:**
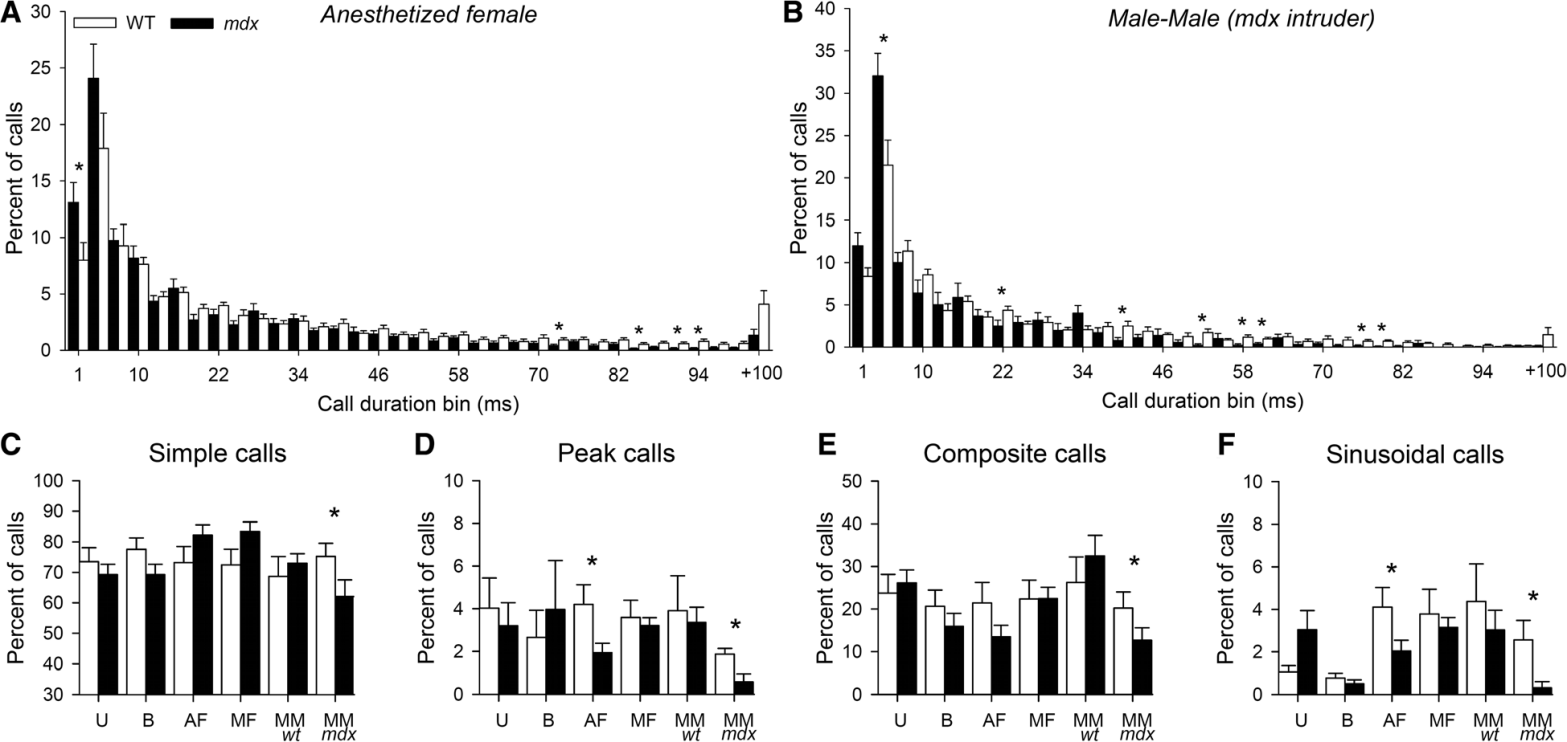
Ultrasonic vocal repertoire in adult mice. **a**, **b** Frequency distribution of ultrasonic vocalizations (%) as a function of call duration (ms) during exposure to anesthetized females (**a**) and to *mdx* male intruders (**b**). **c**–**f** Quantification of distinct call patterns showing context-specific genotype differences in the proportion of simple (**c**), peak (**d**), composite (**e**), and sinusoidal calls (**f**). Call structure was particularly altered during confrontation with anesthetized females and *mdx* male intruders (both parameters; *p* < 0.05). *U* urine test, *B* bedding test, *AF* anesthetized female test, *MF* male–female interaction, *MMwt* interaction with a WT male, *MMmdx* interaction with an *mdx* male. *p < 0.05

Because the large repertoire of mouse ultrasonic vocalizations (Fig. 1) includes syllables of highly variable durations (from <10 to 270 ms), we analyzed the distribution of call durations to determine whether the reduced call duration in *mdx* mice (Fig. 6c) was due to the use of distinct call subtypes. Indeed, the distribution of call durations selectively differed between genotypes during exposure to anesthetized females (*p* < 0.005; Fig. 7a) and *mdx* intruder males (*p* < 0.005; Fig. 7b), as *mdx* residents produced more calls of a short duration and less calls of a long duration (*p* < 0.05). Hence, this qualitative change in the vocal repertoire in *mdx* mice, confirmed by qualitative changes in call structure in the same experimental contexts (see Fig. 7c–f), suggests that *mdx* and WT mice indeed used a distinct repertoire of syllables in response to specific social stimuli.

## Discussion

This study provides the first evidence of abnormal social behavior and communication in the *mdx* mouse model of DMD, which presents a nonsense point mutation leading to complete loss of the full-length dystrophin protein. This supports the hypothesis that *dmd* gene mutations may contribute to the emergence of autistic traits in this syndrome. It is noteworthy that stereotypic behaviors often observed in mouse models of autism, such as altered patterns of grooming activity or repetitive jumping [32], were not a feature of the *mdx* mouse phenotype. However, stereotypies are not systematically detected in mouse models of ASD (e.g., [33]) and the entirety of the cardinal symptoms of ASD is not always expressed in autistic patients [7]. Repetitive behaviors have not been reported in DMD/BMD patients with ASD, suggesting that these syndromes are not associated with stereotypies. Here, we show that *mdx* mice display context-specific behavioral and acoustic alterations, which are likely influenced by the degree of executive/cognitive demand and by emotional and conative factors.

### Context-specific alterations in social behavior

The context-specific changes in social behavior in *mdx* mice suggest specific defects in the behavioral functions involved in the processing of socially relevant information, which may include alterations in emotional reactivity, aggressiveness, and executive functioning. While in the three-chambered test, there were no deficits in sociability, social novelty preference, and short-term memory of social stimuli, during reciprocal interactions in a novel environment, *mdx* mice exhibited altered social behavior. The main difference between these two paradigms is that in the former, intruders are spatially confined and thus behaviorally restricted, while in the latter, direct social encounters require organization of sequences of actions to adapt rapidly to the unpredicted behavior of the intruder mouse. In this situation, *mdx* mice displayed reduced number of contacts with females or with *mdx* male intruders, while the number of contacts with WT intruders was conversely increased, suggesting alterations in adaptive behaviors and thus in executive functions.

Interestingly, when *mdx* mice were confronted with an intruder in their home cage they displayed an increased freezing response selectively during physical contacts, while they went back to normal exploratory activity upon interruption of social contact. This suggested that enhanced fear-related responses could also contribute to the altered social behavior in *mdx* mice [34, 35]. This was associated with an increased number of dominant acts and pursuits initiated by control intruders towards *mdx* residents, suggesting that enhanced emotional reactivity in *mdx* mice might have influenced social hierarchy and/or aggressiveness during interactions with WT mice. However, fear-related responses were not observed in all experimental conditions. Moreover, *mdx* social behavior also varied depending on intruders’ trait behavior and genetic background (e.g., low versus highly aggressive genetic backgrounds). Such context-specific disturbances further support that executive functions enabling adaptation of behavior to distinct contexts and different types of intruders are affected in *mdx* mice.

#### Altered ultrasonic communication

The study of ultrasonic communication in mice is a useful means to unveil phenotypic traits in mouse models of diverse speech and neurodevelopmental disorders including autism [27, 36–38]. Mouse USVs consist in different syllable types produced in bouts that follow a song-like structure, based on call-pattern mechanisms similar to those observed in primates, thus providing a mammalian model to study vocal learning and human disorders associated with speech or communication impairments [39]. Mice normally produce complex ultrasonic vocalizations in response to various sexual and social stimuli [25, 37], which is interpreted as a communicative behavior enabling mother mice to retrieve their pups after removal from the nest during the postnatal period and modulating mice social interactions in adulthood [40, 41]. Here, we show that the production of isolation-induced USVs progressively increases from PND3 to PND9 in both *mdx* and WT pups, as expected [24]. However, transient alterations were observed in *mdx* pups at specific postnatal ages, as also demonstrated in other autism-relevant mouse models [24, 42]. First, there was an abnormal increase in call peak frequency in *mdx* pups at PND3. According to playback experiments [43], mother mice may show a stronger response towards a 65–45 kHz signal than to a 75–55 kHz signal, perhaps because high frequencies propagate less than low frequencies, suggesting that high peak frequencies decrease the functional value of pups “alarm” calls. The second alteration found in *mdx* pups was a change in the frequency modulation patterns at PND3–4 and PND6–7. Postnatal development was associated with a progressive increase in the proportion of highly frequency-modulated calls, such as sinusoidal (i.e., complex) and composite (i.e., frequency jump) calls, and such frequency modulations are believed to be critical for maternal behavior [44]. However, *mdx* pups emitted less “simple” downward calls at PND3 and peak calls at PND6–7, while they displayed more composite calls at PND4 compared to WT mice. This suggests that neonate *mdx* mice use a more complex repertoire of calls compared to WT mice. Interestingly, such changes in mice vocalization repertoire have been previously associated with changes in emotional reactivity or arousal and are consistent with the profile of communication alterations in other rodent models of autism [24, 40].

Context-specific alterations in USVs were also found in adult *mdx* mice and were characterized by a selective reduction in the quantity of vocalizations emitted in presence of anesthetized females and during interactions with other *mdx* male mice. A more modest and non-significant reduction in call rate was also observed during interaction with freely moving females. In contrast, the USVs emitted in low-noise situations in response to potent volatile sexual olfactory stimuli (proestrous-estrous female urine on a cotton swab) or to a combination of volatile and non-volatile sexual stimuli (exploration of female cage bedding) did not reveal major changes in call rate and duration, suggesting that main and accessory olfactory systems are unaltered in *mdx* mice [45, 46]. Also, a putative influence of motor or muscular defects is unlikely. Indeed, the intrinsic laryngeal muscles involved in vocal production by controlling the position and tension of the vocal folds are spared from the dystrophic process in the *mdx* mouse [47]. Although a deficient motor coordination in *mdx* mice [48] could alter the genesis of USVs, as suggested by studies in the Foxp2-deficient mouse [36, 49], this could not explain the selectivity of the deficits in *mdx* mice. Moreover, the recordings performed in presence of an anesthetized female enabled USV analysis in low background noise and ruled out possible biases due to the putative production of USVs by intruders. Our data therefore show that USVs are specifically affected in *mdx* mice in social contexts and more likely depended on altered social motivation.

The USVs normally emitted in the presence of females are thought to facilitate approach behavior and copulation [50], while during male–male encounters they have been associated with expression of affiliative behaviors [25]. When *mdx* mice were submitted to dyadic interactions with either WT females or *mdx* male intruders in a novel environment, the reduced number of vocalizations was associated with a reduced number of contacts initiated by *mdx* residents. Conversely, a slight increase in call rate was associated with an increased number of contacts initiated by *mdx* residents towards WT male intruders. In these situations *mdx* calls had shorter durations and amplitudes, two parameters remarked as prosodic elements conveying critical emotional or motivational information for social interactions [51]. Accordingly, call duration has been shown to be modulated by social motivation [25] and reduced in mouse models of autism [27]. Here, we also demonstrate that adult *mdx* mice used an abnormal vocal repertoire in specific social contexts, i.e., a reduced expression of peak and composite calls in presence of anesthetized females and of sinusoidal and simple calls in male–male encounters. The composition of an adult mouse repertoire normally depends on the context and previous social experience [25]. Short and composite calls are considered as “basic” calls found in many behavioral context whereas upward, frequency jump, u-shape, flat, and chevron (i.e., peak) calls are predominantly found in social situations and therefore considered as more “informative” of the behavioral, emotional, or motivational content of these specific situations. Here, the altered vocal repertoire during male–male encounters was associated with a reduction in the number of contacts, suggesting a functional link between social behavior deficits and altered USVs in *mdx* mice. The selectivity of the observed deficits likely depended on a change in social motivation. This might reflect an altered neural control of call production, perhaps due to the lack of Dp427 in neocortical principal neurons [52], in particular in the anterior cingulate cortex, which takes part in the volitional circuits associated with call onset [38]. Interestingly, some models of autism such as neuroligin-deficient mice have been shown to exhibit deficits in the production of complex calls during social interactions [53], while they were able to produce these calls in other contexts, which also suggests that the altered use of the vocal repertoire may reflect a deficit in behavioral responsiveness to social stimuli eliciting USVs [38].

#### Relevance to DMD and ASD

We show for the first time the presence of context-specific disturbances in social behavior and ultrasonic communication in the dystrophin-deficient *mdx* mouse. Hence, mutations selectively affecting the expression of the full-length Dp427, a common genetic alteration in all DMD patients, appear to be sufficient to significantly alter social behavior and communication. This is in agreement with a case study reporting ASD in a DMD patient holding a mutation that selectively impairs Dp427 expression [15]. Mutations that affect expression of the other C-terminal brain forms of dystrophin have been associated with mental retardation. Therefore, the general observation of social behavior deficits in DMD patients that do not display mental retardation also supports a role for Dp427 in social behavior [10]. Nevertheless, patients with intellectual quotients in the normal range may exhibit deficits in executive functions [3], which might contribute to the emergence of both social behavior and communication deficits.

In *mdx* pups, transient alterations in USVs are readily detectable during the first postnatal week, while social behavior deficits in the adults are associated with the use of an abnormal vocal repertoire of vocalizations in specific social contexts, suggesting alterations in the executive control of social motivation, adaptive behavior and communication. Strikingly, delayed speech development, limited expressive and receptive vocabulary, reduced verbal fluency, and verbal short-term working memory have been described in DMD patients [54, 55] and associated with alterations in adaptive and social skills [10, 12]. Even when ASD is not diagnosed, DMD patients may display moderate weaknesses in social behavior and communication, including language difficulties, tendency of being withdrawn, avoidance of eye contact, difficulties in interpreting facial affect, and problems with theory of mind [56]. Social behavior relies on the functional integrity of the prefrontal cortex and cerebellum [57, 58]. Dysfunctional cortical-cerebellar circuits have been associated with autism [59], and this has also been proposed as a neural basis of the defective social and executive functions in DMD patients [2]. While impairments in executive functions have not been clearly characterized in the *mdx* mouse, this model displays memory deficits, altered synaptic plasticity and enhanced fearfulness [34, 60], suggesting that both cognitive and conative disturbances due to Dp427 loss could contribute to the social behavior and USV alterations.

The mechanisms responsible for emergence of autistic symptoms in DMD patients are still unclear, although one likely hypothesis is an alteration of the molecular interactions between the dystrophin complex and the trans-synaptic neurexin-neuroligin complex in central inhibitory synapses [18]. Dystrophin is selectively involved in the organization of central inhibitory postsynaptic scaffolds by contributing to the recruitment of neuroligin-2 (NLGN2) [19, 61, 62], a synaptic cell adhesion protein involved in structural remodeling of connectivity networks which has been proposed as a candidate gene for ASD [63]. Synaptic molecular alterations specifically due to Dp427 loss in *mdx* mice are characterized by a delocalization of NLGN2 and presynaptic vesicular GABA transporter (VGAT) and a reduction of NLGN2 colocalization with alpha-1 subunit-containing GABA_A_ receptors within hippocampal synaptic layers, suggesting a modification of the molecular mechanisms that normally underlie precise spatio-temporal pattern of GABAergic transmission [61]. Dystrophin loss also alters synapse ultrastructure, clustering of distinct subtypes of GABA_A_ receptors, central inhibitory function, and synaptic plasticity, which has been associated with disturbances in fear-related behaviors, anxiety, and cognitive functions [34, 64–66]. Interestingly, rodent models overexpressing NLGN2 display deficits in reciprocal social interactions and stereotypies [67, 68], while in the knockout model, a reduced production of isolation-induced calls has been reported in pups [69], but no overt impairment in social behavior was found in the adult. Future pharmacological approaches aimed at compensating altered GABAergic function or restoring dystrophin function in *mdx* mice might be helpful to further delineate the physiopathology of autistic traits in DMD, to highlight the importance of the neuroligin and neurexin complex in ASD and to unveil new leads to alleviate behavioral symptoms in DMD patients.

## Conclusions

Our study provides the first evidence that mutations impeding expression of the brain full-length dystrophin affect social behavior and communication. The deficits displayed by *mdx* mice support the hypothesis that altered social cognition may confer vulnerability to autism in DMD. The context-specific changes in social behavior and communication in *mdx* mice likely depend on the different behavioral/emotional demands associated with specific test conditions. How such alterations may contribute to the occurrence of ASD in restricted subpopulation of DMD patients remains to be determined. In ASD, only rare cases can be ascribed to single-gene defects, while multiple interacting genetic factors appear to be the main causative determinants of “idiopathic” autism, and epigenetic/environmental factors may also contribute to the variable expression of autism-related traits [70–72]. This multi-hit hypothesis likely applies to the DMD condition, in which a full autistic-like phenotype would only be achieved in some patients when the mutation in the *dmd* gene alters expression of additional C-terminal dystrophin products [73] and/or when additional mutations or copy number variations (CNVs) in other genes disturb expression of other synaptic proteins, leading to cumulative deficits that would then meet criteria for ASD diagnosis [14, 74, 75]. Therefore, correlations between the patient’s genotype and presence of autism should be systematically analyzed in DMD patients and could include searches for additional genomic abnormalities beyond *dmd* gene mutations. Conversely, specific mutations in the *dmd* gene lead to late onset and mild muscular phenotypes, as illustrated in patients cases with such mutations that only displayed mental retardation or autistic-like behaviors [14, 76], and some of these genetic alterations likely have been missed or underestimated in former genome-wide studies of patients with idiopathic autism [75, 77]. Therefore, higher resolution mutation screening of the *DMD* gene in ASD patients should be encouraged.

## Additional files

Additional file 1:
**Supplementary method section**. Semi-automatic classification of mouse USVs. (PDF 144 kb) Additional file 2: Figure S1. Exploration of female olfactory stimuli. (DOC 317 kb) Additional file 3:
**Supplementary result section**. Correlation analyses. (PDF 37 kb)

## Abbreviations

ANOVA: analysis of variance
ASD: autism spectrum disorders
BMD: Becker muscular dystrophy
CNVs: copy number variations
DMD: Duchenne muscular dystrophy
Dp427: 427-kDa cytoskeleton-associated dystrophin protein
FFT: fast Fourier transform
GABA: gamma-aminobutyric acid
IP: intraperitoneal
KS: Kolmogorov-Smirnov
NLGN1–4: neuroligin family of synaptic cell adhesion proteins
Pamp: call peak amplitude
Pfreq: call peak frequency
PND: postnatal day
USV: ultrasonic vocalization
VBA: visual basic for applications
VGAT: presynaptic vesicular GABA transporter
WT: wild type
