# Supplementary material for: Altered social behavior and ultrasonic communication in the dystrophin-deficient mdx mouse model of Duchenne muscular dystrophy
Source: Mol Autism. 2015 Oct 29;6:60. doi: 10.1186/s13229-015-0053-9 (PMC4627616; doi:10.1186/s13229-015-0053-9)
Supplement: Additional file 1: — Supplementary method section. Semi-automatic classification of mouse USVs. (PDF 144 kb) [file 13229_2015_53_MOESM1_ESM.pdf]

Miranda R et al. Altered social behavior and ultrasonic communication in the dystrophin-deficient mdx mouse model of Duchenne muscular

Additional file 1: Supplementary method section: Semi-automatic classification of mouse USVs.

Automatic identification of call categories

Identification of call categories is performed combining custom VBA-based macros and visual inspection of sonograms. Using Avisoft SASLab Pro v4.40, an automatic threshold-based detection of calls is first performed by extracting time/frequency contour data every 10 ms for each call (Figure 1).

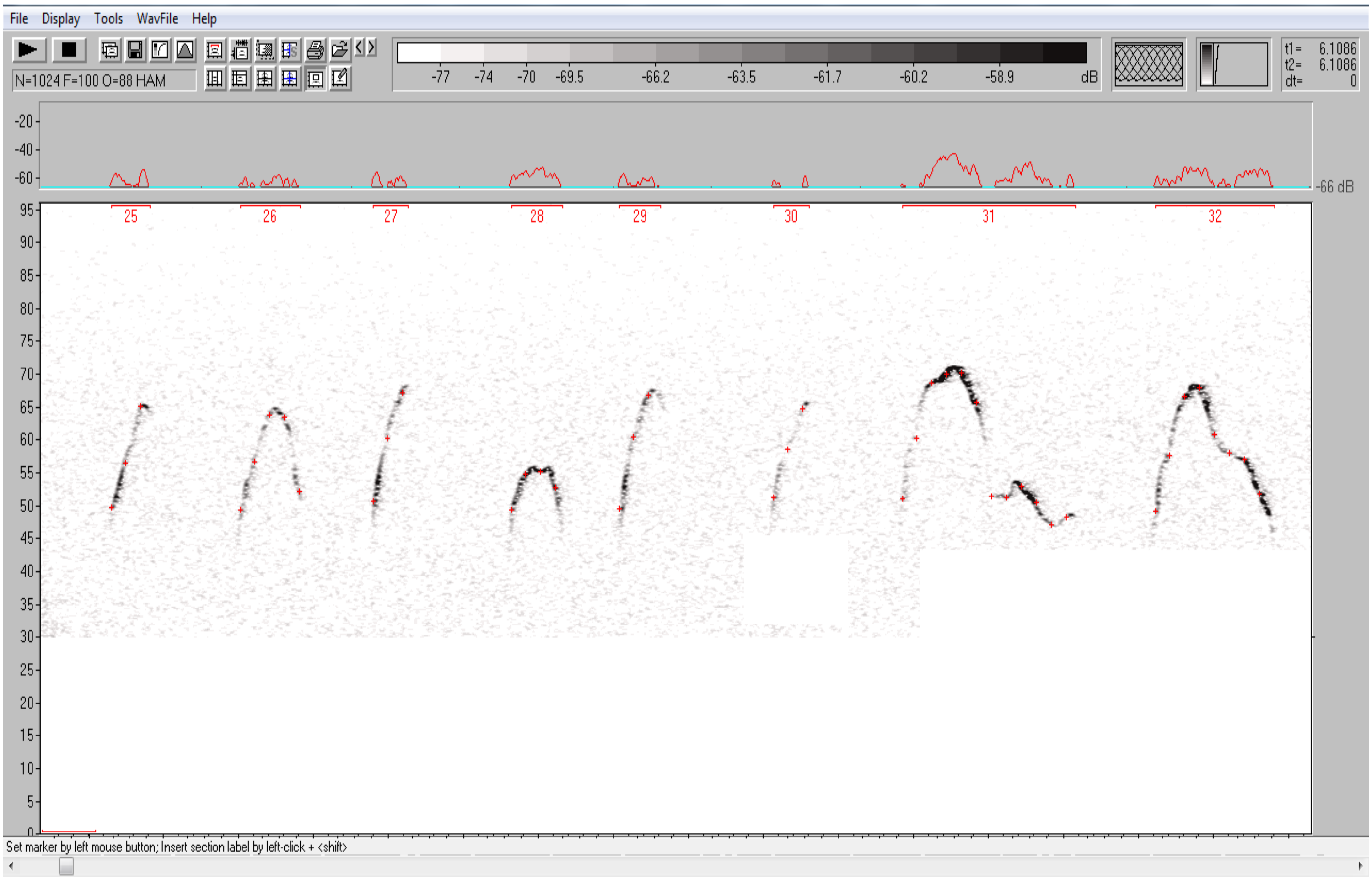

Figure 1. Spectrogram fragment including a total of 8 calls showing the location of frequency data points (red crosses) taken every 10 ms within each call.

Data is then imported into Excel software (Microsoft corp.) including call number (#), duration (s), start and end time and peak frequency values taken every 10 ms for each call (Table 1).

Miranda R et al. Altered social behavior and ultrasonic communication in the dystrophin-deficient mdx mouse model of Duchenne muscular

Additional file 1: Supplementary method section: Semi-automatic classification of mouse USVs.

#	Duration (s)	start time	end time	Pf (0ms)	Pf (10ms)	Pf (20ms)	Pf (30ms)	Pf (40ms)	Pf (50ms)	Pf (60ms)	Pf (70ms)	Pf (80ms)	Pf (90ms)	Pf (100ms)	Pf (110ms)
25	0,0253	5,1673	5,1926	49600	56400	65000									
26	0,0393	5,2533	5,2926	49300	56600	63700	63300	52100							
27	0,0226	5,342	5,3646	50600	60100	67100									
28	0,0333	5,434	5,4673	49300	54700	55100	52600								
29	0,0266	5,506	5,5326	49500	60300	66700									
30	0,0233	5,6086	5,632	51100	58500	64600									
31	0,1146	5,6946	5,8093	51000	60100	68600	69900	70100	65600	51300	51100	52800	50400	47000	48100
32	0,0786	5,8633	5,942	49100	57500	66500	67800	60700	57900	57000	51700				

Table 1. Peak frequency (Pf) values at 10 ms intervals for each call (#).

Automatic classification of calls based on the frequency values taken at 10ms-intervals is achieved with the aid of a custom VBA macro. Within each call, each 10-ms interval is assigned to a qualitative value representing ascending, descending or flat changes in frequency (≥1.5 Hz). Short calls are simply identified based on their duration (<10 ms) (Table 2).

#	Duration (s)	Start time	End time	10-0	20-10	30-20	40-30	50-40	60-50	70-60	80-70	90-80	100-90	110-100
25	0,0253	5,1673	5,1926	A	A									
26	0,0393	5,2533	5,2926	A	A	F	D							
27	0,0226	5,342	5,3646	A	A									
28	0,0333	5,434	5,4673	A	F	D								
29	0,0266	5,506	5,5326	A	A									
30	0,0233	5,6086	5,632	A	A									
31	0,1146	5,6946	5,8093	A	A	F	F	D	D	F	A	D	D	F
32	0,0786	5,8633	5,942	A	A	F	D	D	F	D				

Table 2. Conversion of frequency differences between consecutive data points into a qualitative category (Ascending (A): positive difference >1.5 kHz, Descending (D): negative difference >1.5 kHz, Flat (F): differences <1.5 kHz, Short: if duration <10 ms)

Then, to determine the category of each call (upward, downward, flat, peak, U-shaped or sinusoidal) the macro integrates the 10-ms sequences of qualitative information within the total duration of a call based on a set of logical-based possible combinations between 'first order categories' (i.e., ascending, descending, flat) and 'second order categories' (i.e., peak, U-shaped, sinusoidal) (Tables 3-5).

Additional file 1: Supplementary method section: Semi-automatic classification of mouse USVs.

	A	D	F
A	AA (**A**)	AD (**P**)	AF (**A**)
D	DA (**U**)	DD (**D**)	DF (**D**)
F	FA (**A**)	FD (**D**)	FF (**F**)
P	PA (**S1L**)	PD (**P**)	PF (**P**)
U	UA (**U**)	UD (**S1R**)	UF (**U**)
S1R	**S2D**	**S1R**	**S1R**
S1L	**S1L**	**S2U**	**S1L**
S2D	**S2D**	**S2D1U**	**S2D**
S2U	**S2U1D**	**S2U**	**S2U**

Table 3. Main logical combinations of first and second order qualitative categories of frequency modulations to deduce the definitive call categories. The resulting categories are shown between brackets and/or in red for sinusoidal waveforms. Al categories highlighted in red can be unified as sinusoidal. (A, ascending; D, descending, P, Peak; U, U-shaped, S1R, sinusoidal with one peak on the right; S1L, sinusoidal with one peak on the left; S2D, sinusoidal with two inverted peaks; S2U, sinusoidal with two upper peaks; S2D1U, sinusoidal with two inverted peaks plus one upper peak; SU1D, sinusoidal with two upper peaks plus one inverted peak)

#	duration	start time	end time	0-20	20-30	30-40	40-50	50-60	60-70	70-80	80-90	90-100	100-110	Definitive category
25	0,0253	5,1673	5,1926	A										Ascending
26	0,0393	5,2533	5,2926	A	A	P								Peak
27	0,0226	5,342	5,3646	A										Ascending
28	0,0333	5,434	5,4673	A	P									Peak
29	0,0266	5,506	5,5326	A										Ascending
30	0,0233	5,6086	5,632	A										Ascending
31	0,1146	5,6946	5,8093	A	A	A	P	P	P	S1L	S2U	S2U	S2U	Sinusoidal
32	0,0786	5,8633	5,942	A	A	P	P	P	P					Peak

Table 4. Logical conversion of 'first order categories' (i.e., ascending, descending and flat) into 'second order' categories.

#	Sequential logical-based combinations	Result
25	A + A → A	Ascending
26	A + A → A + F → A + D → P	Peak
27	A + A → A	Ascending
28	A + F → A + D → P	Peak
29	A + A → A	Ascending
30	A + A → A	Ascending
31	A + A → A + F → A + F → A + D → P + D → P + F → P + A → S1L + D → S2U + D → S2U + F → S2U	Sinusoidal
32	A + A → A + F → A + D → P + D → P + F → P + D → P	Peak

Table 5. Example of the sequential logical-based combinations required to achieve definitive call categories.

Automatic threshold detection is not accurate to identify unstructured, harmonic and frequency jump (i.e., composite) calls, or in conditions of low signal to noise ratio. In such conditions, an accurate classification requires visual inspection of spectrograms and manual identification of calls using for example the *interactively (section labels)* function of Avisoft software. Data derived from manual inspection of sonograms are transferred into Excel software and combined with previous automatically classified data with the aid of a second VBA-

based custom macro. Using the start points of each call as a reference, this second macro overwrites and corrects previous erroneous automatic classification data with manually-defined categories when appropriate.
