# Supplementary material for: Altered social behavior and ultrasonic communication in the dystrophin-deficient mdx mouse model of Duchenne muscular dystrophy
Source: Mol Autism. 2015 Oct 29;6:60. doi: 10.1186/s13229-015-0053-9 (PMC4627616; doi:10.1186/s13229-015-0053-9)
Supplement: Additional file 2: Figure S1. — Exploration of female olfactory stimuli. (DOC 317 kb) [file 13229_2015_53_MOESM2_ESM.doc]

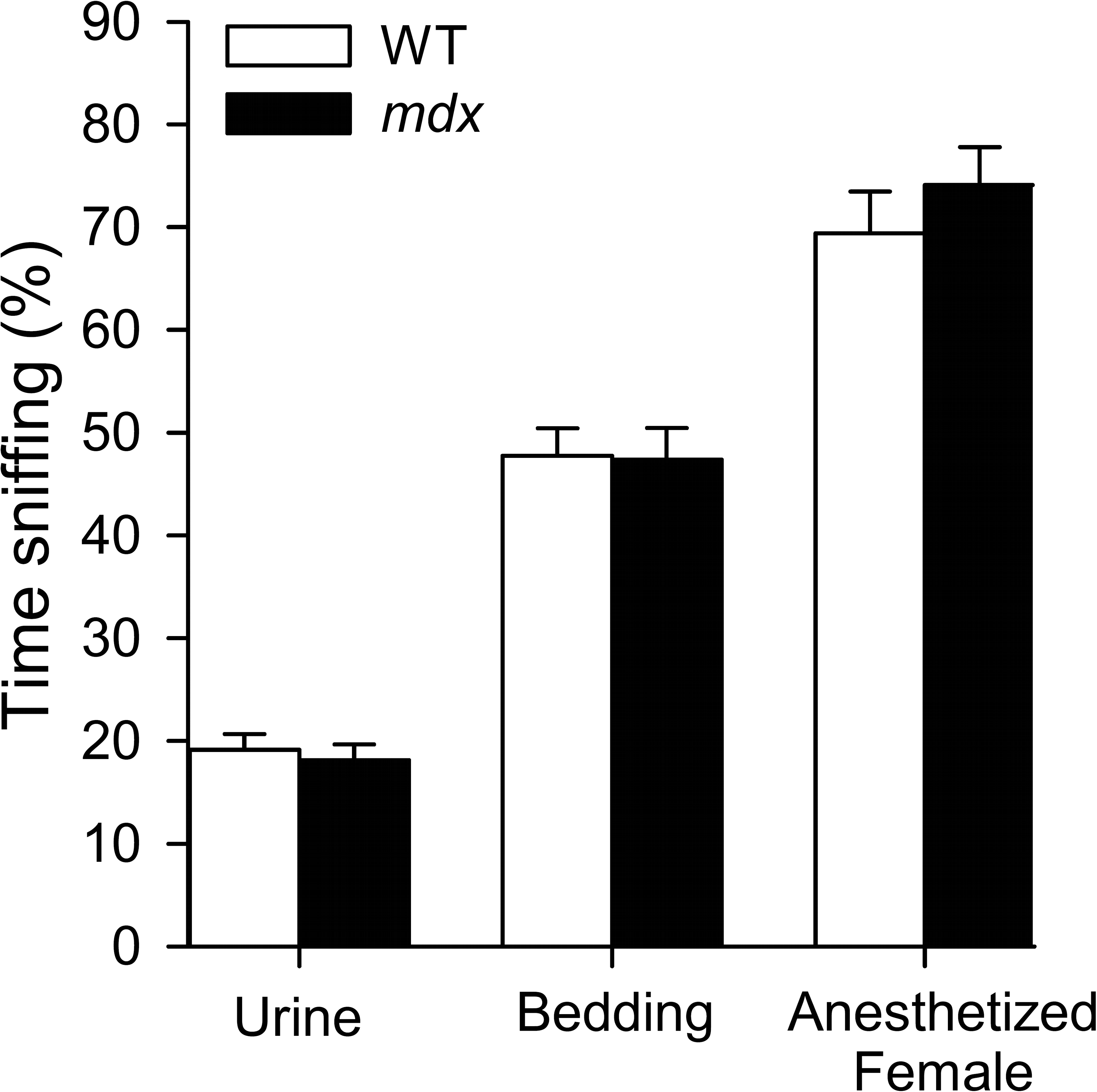


**Figure S1.** *Exploration of female olfactory stimuli (urine and cage bedding) and anesthetized females*. Wild-type (WT) (white bars) and *mdx* (black bars) male mice spent comparable time sniffing the stimuli, and both genotypes were more attracted by the anesthetized female than by female cage bedding and urine.
