# Supplementary material for: Altered social behavior and ultrasonic communication in the dystrophin-deficient mdx mouse model of Duchenne muscular dystrophy
Source: Mol Autism. 2015 Oct 29;6:60. doi: 10.1186/s13229-015-0053-9 (PMC4627616; doi:10.1186/s13229-015-0053-9)
Supplement: Additional file 3: — Supplementary result section. Correlation analyses. (PDF 37 kb) [file 13229_2015_53_MOESM3_ESM.pdf]

Miranda R et al. Altered social behavior and ultrasonic communication in the dystrophin-deficient mdx mouse model of Duchenne muscular

Additional file 3: Supplementary results: Correlation analyses.

1. Correlations in distinct experimental contexts

Relationship between the amount of exploration of social stimuli and USVs production in distinct experimental contexts, as presented in Figure 6B in the paper, shows that USV production was strongly modulated by the social context. However, long time spent vocalizing was not systematically associated with long time spent investigating social stimuli across tests (see legend of Figure 6B). As shown in the table below, there was no significant correlation between percent time sniffing and percent time vocalizing across tests in both genotypes. However, note that *mdx* mice displayed inverse correlations as compared to WT mice when confronted to female bedding and during encounters with *mdx* male intruders (highlighted in bold letters).

TASK	Genotype	Correlation
URINE	WT	,523
	MDX	,846
BEDDING	WT	**,716**
	MDX	**-,511**
ANESTHETIZED	WT	,224
	MDX	,527
MALE-FEMALE	WT	-,606
	MDX	-,075
MALE-WT	WT	-,119
	MDX	,411
MALE-MDX	WT	**-,635**
	MDX	**,696**

Percent time sniffing vs percent time vocalizing (all p>0.05; N.S.)

2. Detailed correlations in response to social stimuli

As shown in the tables below, during exposure to olfactory stimuli and anesthetized females, call amplitude (intensity) correlated with the number of sniffing episodes in WT mice (positive correlation in bedding and anesthetized female tasks, negative correlation in the urine task). In contrast, no significant correlation was found between behavioral and acoustic variables in *mdx* mice in these tasks.

Miranda R et al. Altered social behavior and ultrasonic communication in the dystrophin-deficient mdx mouse model of Duchenne muscular

Additional file 3: Supplementary results: Correlation analyses.

ANESTHETIZED FEMALE

Genotype		Sniffing episodes	Time sniffing (%)
WT	Call rate	,590	-,130
	Call duration	,245	-,159
	Pfreq(st)	,181	-,007
	Pfreq(end)	,292	,036
	Pamp(st)	,536	-,289
	Pamp(end)	**,708(*)**	-,415
MDX	Call rate	,209	-,220
	Call duration	-,066	,146
	Pfreq(st)	-,183	-,065
	Pfreq(end)	-,094	-,064
	Pamp(st)	,325	-,220
	Pamp(end)	**,284**	-,321

*** Correlation is significant at the 0.05 level (2-tailed).**

FEMALE BEDDING

Genotype		Sniffing episodes	Time sniffing (%)
WT	Call rate	-,508	,412
	Call duration	-,182	,291
	Pfreq(st)	,067	-,019
	Pfreq(end)	,291	-,394
	Pamp(st)	,481	,055
	Pamp(end)	**,819(*)**	-,677
MDX	Call rate	,131	-,336
	Call duration	,454	-,474
	Pfreq(st)	,467	-,302
	Pfreq(end)	,478	-,097
	Pamp(st)	-,258	,253
	Pamp(end)	**-,162**	,164

FEMALE URINE

Genotype		Sniffing episodes	Time sniffing (%)
WT	Call rate	-,040	-,474
	Call duration	-,466	,693
	Pfreq(st)	-,328	-,381
	Pfreq(end)	,336	-,040
	Pamp(st)	**-,916(*)**	-,349
	Pamp(end)	-,788	-,564
MDX	Call rate	,212	,022
	Call duration	,300	,114
	Pfreq(st)	-,058	-,136
	Pfreq(end)	-,584	,261
	Pamp(st)	**,145**	-,012
	Pamp(end)	,211	-,030

Additional file 3: Supplementary results: Correlation analyses.

3. Detailed correlations during forced encounters with intruders

During social interactions (tables below), call rate was positively correlated with the number of pursuits performed by WT residents confronted with WT intruders. However, these parameters were uncorrelated in *mdx* residents. The number of dominant acts was positively correlated with call frequency and negatively correlated with call duration in WT mice, whereas in *mdx* mice dominant acts were only positively correlated with call amplitude (intensity).

MALE VS. WT INTRUDERS

Resident genotype		Contacts (n)	Pursuits (n)	Dominant acts (n)
WT	**Call rate**	,508	**,682(*)**	-,300
	**Call duration**	,002	,270	**-,847(**)**
	**Pfreq(st)**	,369	,109	,638
	**Pfreq(end)**	,553	,333	**,848(**)**
	**Pamp(st)**	,138	,159	-,497
	**Pamp(end)**	-,390	-,361	**-,614**
MDX	**Call rate**	,451	**,245**	,556
	**Call duration**	,478	,413	**,696**
	**Pfreq(st)**	-,514	-,355	-,601
	**Pfreq(end)**	-,260	-,022	**-,282**
	**Pamp(st)**	,712	,699	,582
	**Pamp(end)**	,561	,643	**,815(*)**

** Correlation is significant at the 0.01 level (2-tailed).
* Correlation is significant at the 0.05 level (2-tailed).

In encounters with *mdx* intruders (below), measures in WT mice were uncorrelated whereas for *mdx* residents call frequency was negatively correlated with the number of contacts and pursuits.

MALE VS. MDX INTRUDER

Resident genotype		Contacts (n)	Pursuits (n)	Dominant acts (n)
WT	**Call rate**	,203	,196	,133
	**Call duration**	,229	,650	,695
	**Pfreq(st)**	**-,597**	**-,012**	-,353
	**Pfreq(end)**	-,390	-,338	-,538
	**Pamp(st)**	-,363	,030	-,273
	**Pamp(end)**	-,428	-,055	-,465
MDX	**Call rate**	-,005	,291	,221
	**Call duration**	,103	,315	,311
	**Pfreq(st)**	**-,672(*)**	**-,700(*)**	-,491
	**Pfreq(end)**	-,464	-,512	-,502
	**Pamp(st)**	-,350	,218	,415
	**Pamp(end)**	-,656	-,459	-,399

* Correlation is significant at the 0.05 level (2-tailed).

Additional file 3: Supplementary results: Correlation analyses.

In male-female encounters (below) all variables in WT and *mdx* mice were not significantly correlated.

MALE VS. FEMALE

Resident genotype		Contacts (n)	Time in contact (%)	Latency 1st contact
WT	Call rate	,559	,306	-,071
	Call duration	,476	,266	,064
	Pfreq(st)	-,284	-,420	-,186
	Pfreq(end)	,061	-,102	-,452
	Pamp(st)	,260	-,032	,318
	Pamp(end)	,176	-,324	-,110
MDX	Call rate	-,228	-,552	-,061
	Call duration	-,208	-,465	-,034
	Pfreq(st)	-,271	-,378	,069
	Pfreq(end)	-,361	-,501	,101
	Pamp(st)	,402	,513	-,338
	Pamp(end)	,456	,440	-,416

4. Conclusion

Several significant correlations detected in the WT mice were not found in *mdx* mice. This suggests a disorganization of the relationships between social behavior and ultrasonic communication in *mdx* mice.
